# Atmospheric CO_2_ levels from 2.7 billion years ago inferred from micrometeorite oxidation

**DOI:** 10.1126/sciadv.aay4644

**Published:** 2020-01-22

**Authors:** O. R. Lehmer, D. C. Catling, R. Buick, D. E. Brownlee, S. Newport

**Affiliations:** 1 Department of Earth and Space Sciences, University of Washington, Seattle, WA 98195, USA.; 2 Astrobiology Program, University of Washington, Seattle, WA 98195, USA.; 3 MS 239-4, Space Science Division, NASA Ames Research Center, Moffett Field, CA 94035, USA.; 4 Department of Astronomy, University of Washington, Seattle, WA 98195, USA.

## Abstract

Earth’s atmospheric composition during the Archean eon of 4 to 2.5 billion years ago has few constraints. However, the geochemistry of recently discovered iron-rich micrometeorites from 2.7 billion–year–old limestones could serve as a proxy for ancient gas concentrations. When micrometeorites entered the atmosphere, they melted and preserved a record of atmospheric interaction. We model the motion, evaporation, and kinetic oxidation by CO_2_ of micrometeorites entering a CO_2_-rich atmosphere. We consider a CO_2_-rich rather than an O_2_-rich atmosphere, as considered previously, because this better represents likely atmospheric conditions in the anoxic Archean. Our model reproduces the observed oxidation state of micrometeorites at 2.7 Ga for an estimated atmospheric CO_2_ concentration of >70% by volume. Even if the early atmosphere was thinner than today, the elevated CO_2_ level indicated by our model result would help resolve how the Late Archean Earth remained warm when the young Sun was ~20% fainter.

## Teaser

Iron-rich micrometeorites from a 2.7 billion-year-old limestone could constrain atmospheric CO_2_ levels on the early Earth.

## INTRODUCTION

The atmospheric CO_2_ concentration of the Archean Earth is highly uncertain. In the Archean, the Sun was 20 to 30% less luminous and CO_2_ levels would have needed to be much higher than modern to maintain a climate suitable for liquid water, perhaps by a factor of 10^2^ to 10^3^, depending on the concentration of other greenhouse gases, such as CH_4_ [e.g., (*1*, *2*)]. In addition to atmospheric models, Archean paleosols and other proxies have been examined to constrain atmospheric CO_2_ levels. Estimates from these studies range between ~3 × 10^−3^ and ~0.75 bar of CO_2_ during the Archean [see section 11.4.3 of (*3*) for a review]. Thus, the estimated atmospheric CO_2_ level in the Archean spans ~3 orders of magnitude.

The spread in the Archean atmospheric CO_2_ concentrations is part of a debate about the climate of early Earth. Isotopic analyses of phosphates (*4*) and deuterium (*5*) suggest that the Archean surface temperature was temperate and <40°C. In addition, the presence of Archean glacial deposits [e.g., (*6*)] indicates early Earth was at least periodically cool. However, a high-temperature Archean with surface temperatures of 70 ± 15°C has been proposed on the basis of measurements of oxygen isotopes in Archean cherts (*7*). The possible presence of a low-viscosity Archean ocean (*8*) and the thermostability of resurrected Archean proteins (*9*) are also used for claiming a high-temperature Archean. Additional CO_2_ estimates for the Archean atmosphere could help resolve this temperature uncertainty and provide insight into the conditions for life on early Earth.

It was recently proposed that Archean, spherical, iron-rich (type I) micrometeorites could have been oxidized by modern levels of O_2_ in the upper atmosphere (*10*, *11*). However, very low levels of O_2_ in the Archean atmosphere inferred from a large variety of proxies [e.g., (*12*)] motivate considering oxidation by CO_2_ as an alternative (*13*). Thus, the micrometeorites could provide a new constraint on Archean CO_2_ levels.

When FeNi metal micrometeoroids enter the atmosphere at hypervelocity, they can melt, and while molten, they react readily with the surrounding atmosphere (*14*). During this reaction, iron micrometeorites exposed to O_2_ or CO_2_ can oxidize some or all of the metal to species such as wüstite [Fe_(1–*x*)_O] and magnetite (Fe_3_O_4_) (*15*). Depending on size, entry speed, and entry angle, the micrometeorites melt and oxidize in the upper atmosphere for only a few seconds at approximately 75 to 90 km above the surface for modern Earth and solidify well before reaching the lower atmosphere. After solidifying in the upper atmosphere, iron-rich micrometeorites become largely inert and can preserve their oxidation state through geologic time (*10*).

The possibility that Archean micrometeorites could provide a proxy for atmospheric composition was first proposed by Tomkins *et al.* (*10*). They found 59 iron-rich micrometeorites in a 2.7 billion–year–old limestone from the Pilbara region of northwestern Australia that contained magnetite, wüstite, and metallic iron. Tomkins *et al.* concluded that micrometeorite oxidation most likely occurred in Earth’s atmosphere and suggested that ~20% atmospheric oxygen in the upper atmosphere was responsible. They discounted CO_2_ in favor of oxidation from atmospheric O_2_ because of slow kinetics in low-temperature (1100 K), high-pressure (1 bar) laboratory measurements of oxidation of solid (and not molten) Fe by CO_2_ and their equilibrium-based calculations, which implied that small, iron-rich micrometeorites would not be oxidized in a low-CO_2_ atmosphere (<10% by volume). They also note that a CO_2_-rich atmosphere could contain some CO, a reductant. However, such an atmosphere should also produce O_2_ and O_3_ [e.g., (*16*)], which could mitigate the reducing capacity of CO. In addition, Fe-rich micrometeorites melt for only a few seconds during entry and likely do not reach equilibrium during this time (*10*), making it premature to rule out CO_2_ as a plausible oxidant.

While an oxygen-rich atmosphere is certainly capable of oxidizing iron micrometeorites, an upper atmosphere of ~20% O_2_ is difficult to reconcile with evidence for an anoxic atmosphere in the Archean where O_2_ was likely less than 10^−4^ bar at altitude (*17*) and less than 1 ppmv (parts per million by volume) at ground level (*12*, *18*–*20*). In addition to proxy data showing low atmospheric O_2_ in the Archean, current understanding of atmospheric mixing does not support an O_2_-rich Archean upper atmosphere with anoxic lower atmosphere. Turbulent mixing, particularly from breaking gravity waves (which physics dictates increase in amplitude with altitude), mixes the major constituents of the atmosphere up to the homopause at ~100-km altitude on modern Earth. Such waves are shed from airflow over surface topography, jets, and thermal tides, which are all processes that would have occurred on ancient Earth.

The homopause is the pressure, or altitude, below which the major atmospheric constituents are well mixed due to turbulence, and on modern Earth is well above the melting altitude for micrometeorites between roughly 65 and 90 km. It is tempting to speculate that the homopause may have occurred at a higher pressure on Archean Earth, and thus at a lower altitude, more consistent with the melting of micrometeorites. However, a high-pressure homopause in the Archean would be physically unusual compared with all other known terrestrial atmospheres. In the Solar System, rocky bodies have homopause pressures of ~10^−2^ Pa on Earth, ~10^−2^ to ~10^−5^ Pa on Mars (depending on season) (*21*), ~10^−3^ Pa on Venus, and ~10^−4^ Pa on Titan [(*3*), p. 6]. Thus, a high-pressure homopause on Archean Earth may be counter to current understanding of atmospheric mixing and available data [see sections 4.3 and 4.4 of (*3*) for a detailed discussion of atmospheric mixing]. Even if the ground-level atmospheric pressure in the Archean were lower than modern (*22*, *23*), the upper atmosphere would still be in approximate hydrostatic equilibrium. So, a lower ground-level pressure would not alter the absolute pressure aloft at which micrometeorites melt (*10*). An Archean model with constant vertical eddy mixing coefficient of 10^5^ cm^2^ s^−1^ (*11*) produces high O_2_ at altitude, but the assumed eddy mixing is an order to orders of magnitude lower than that determined empirically for the upper atmosphere of modern Earth (~10^6^ cm^2^ s^−1^) (*24*) or required to model the thin atmosphere of Mars (e.g., 10^6^ cm^2^ s^−1^ at 20-km to 4 × 10^7^ cm^2^ s^−1^ at 80-km altitude) (*25*).

For all the above reasons, we propose a CO_2_-rich atmosphere capable of oxidizing Fe micrometeorites may have been present at 2.7 Ga. A CO_2_-rich atmosphere is consistent with geochemical analyses of the acid weathering of Archean soils that became paleosols [e.g., (*26*)] and agrees with current models of the Archean carbon cycle and climate (*2*), and demands no peculiar set of circumstances to create high upper atmosphere O_2_ in an anoxic Archean. Furthermore, it has long been demonstrated in the metallurgy industry that CO_2_ can oxidize metallic Fe under various atmospheric conditions and temperatures [e.g., (*27*)].

By modeling the motion, heating, and evaporation of micrometeorites during atmospheric entry, and using a rate constant for Fe oxidation via CO_2_ from laboratory measurements (*28*) (see Materials and Methods for details), we investigate what atmospheric CO_2_ levels could explain the reported oxidation of Archean micrometeorites. In this way, we seek to provide a new constraint on atmospheric CO_2_ levels during the Archean.

## RESULTS

**Fig. 1:**
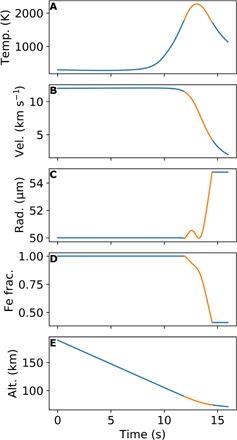
Single model run for a particle with an entry angle of 45° from zenith through a 39% CO_2_, 61% N_2_ atmosphere (50 wt % CO_2_, 50 wt % N_2_). The plots above show (**A**) micrometeorite temperature in kelvin, (**B**) micrometeorite velocity in kilometers per second, (**C**) micrometeorite radius in micrometers, (**D**) mass fraction of metallic Fe to oxidized FeO, and (**E**) micrometeorite’s altitude above Earth’s surface in kilometers. The orange part of each curve indicates the micrometeorite is molten. For plot (C), the micrometeorite increases in radius because the oxide layer is growing faster than it is evaporating. The oxidation of high-density Fe to the lower-density FeO results in a less dense particle and, thus, a larger radius. In this simulation, the micrometeorite is molten for 2.6 s between 11.9 and 14.5 s. The initial radius is 50 μm, the final radius is 54.8 μm, the final Fe fractional mass is 41% (37% by cross-sectional area), and the maximum temperature reached is 2275 K at an altitude of 81.2 km. See the Supplementary Materials for an animation of this figure.

We model 15,000 randomly generated micrometeorites entering a 1 bar N_2_-CO_2_ atmosphere with CO_2_ concentrations between ~2 and ~85% by volume [3 to 90 weight % (wt %)]. We assume that all particles start as pure Fe upon entry. Our model tracks the motion, oxidation, and evaporation of each particle, along with its composition throughout its descent in the atmosphere. We look at the cross section of each simulated micrometeorite and compare the total area of unoxidized Fe to the total cross-sectional area of the particle (i.e., oxidized Fe area plus unoxidized Fe area). We chose to measure the cross-sectional area to make empirical micrometeorite data readily comparable to our model results. Figure 1 shows an example of our model for a single micrometeorite entering a 39% CO_2_, 61% N_2_ atmosphere.

The atmosphere of the Archean was O_2_ poor [e.g., (*29*)]. However, photochemical models of Earth-like planets around Sun-like stars indicate that minor atmospheric O_2_ could be sustained photochemically in the upper atmosphere via CO_2_ photolysis. From such models, the O_2_ level in the upper atmosphere could reach ~1% by volume in a high (90% by volume) CO_2_ atmosphere while remaining a trace gas at the planetary surface (*16*). This O_2_ concentration is well below the modern levels required to explain the oxidation of Archean micrometeorites (*10*).

**Fig. 2:**
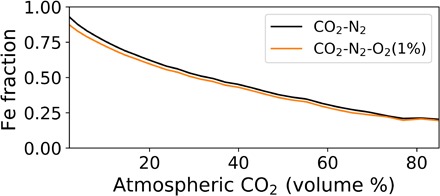
Comparison of unoxidized Fe area in CO_2_-N_2_ and CO_2_-N_2_-O_2_ atmospheres with increasing CO_2_. The black curve shows the mean model cross-sectional area of unoxidized Fe compared with the total cross-sectional area of the micrometeorite for a CO_2_-N_2_ atmosphere. It is the same curve as in Fig. 3. The orange contour shows the same simulated micrometeorites but entering a CO_2_-N_2_-O_2_ atmosphere where the O_2_ represents 1% by volume. The addition of 1% O_2_ to the atmosphere has little impact on the average.

To address the possibility of oxidation by major CO_2_ and minor O_2_ in the Archean, we ran our model with a CO_2_-N_2_-O_2_ atmosphere. Each of the 15,000 modeled micrometeorites was simulated, entering both a CO_2_-N_2_–only atmosphere and a CO_2_-N_2_-O_2_ (1%) atmosphere. As an upper bound, the atmospheric O_2_ was fixed at 1% and did not decrease with altitude in the altitude range for micrometeorite melting. The results of both runs are shown in Fig. 2. The black curve in Fig. 2 shows the mean unoxidized Fe fractional area in our modeled micrometeorites as atmospheric CO_2_ increases for the CO_2_-N_2_ atmosphere, while the orange curve shows the same for micrometeorites entering the CO_2_-N_2_-O_2_ (1%) atmosphere. The difference in final Fe fractional area between the two atmospheres is negligible, especially at high CO_2_ concentrations, showing that CO_2_ dominates as the oxidant when O_2_ is ~1% or lower.

**Fig. 3:**
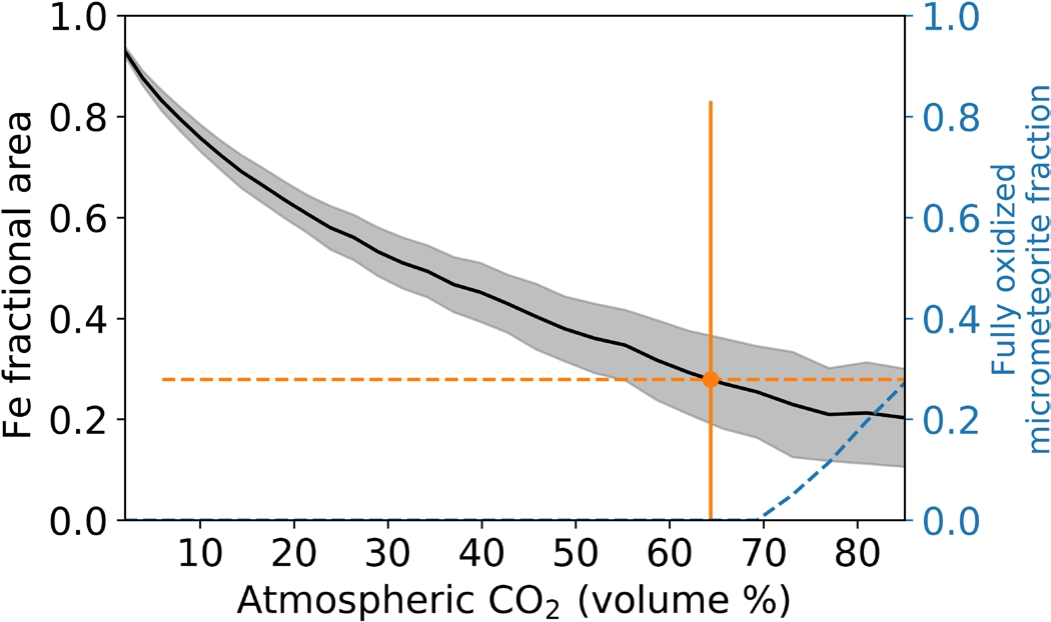
Simulated fractional area of unoxidized Fe with increasing atmospheric CO_2_. The atmosphere was assumed to be composed of pure N_2_ and CO_2_. The black curve shows the mean model prediction for the cross-sectional area of unoxidized Fe compared with the total cross-sectional area of the micrometeorite. The simulated micrometeorites were assumed to have spherical, central metal beads so the cross-sectional area of the unoxidized Fe bead is a maximum. The gray-shaded area shows the 2σ confidence interval of our model. The orange dot and solid error bar show the mean unoxidized Fe fractional area and 2σ confidence interval from the two Fe-FeO micrometeorites reported by Tomkins *et al.* (*10*). The corresponding uncertainty in atmospheric CO_2_ from the Tomkins *et al.* data is shown by the dashed orange error bars. The Tomkins *et al.* data point indicates the CO_2_ level was at $64_{-58}^{+36}$% (2σ). The dashed blue line shows the fraction of modeled micrometeorites that were fully oxidized in the atmosphere with no remaining metallic Fe. Such particles could lead to magnetite-rich micrometeorites and appear in our model once atmospheric CO_2_ reaches ~70%.

The Archean CO_2_ estimates from the modeling are shown in Fig. 3 (the black line of Fig. 3 is the same as in Fig. 2). On average, we find that the unoxidized Fe fractional area of sectioned micrometeorites decreases monotonically as atmospheric CO_2_ concentrations increase. The solid black curve in Fig. 3 shows the mean unoxidized Fe area in our modeled micrometeorites as atmospheric CO_2_ increases. The gray-shaded region is the model 2σ uncertainty arising from uncertainties in initial mass, velocity, and impact angle (see Materials and Methods). The orange dot and error bar show the mean fractional Fe area measured by Tomkins *et al.*

From the black curve in Fig. 3, we estimate an Archean CO_2_ volume mixing ratio of $64_{-58}^{+36}$% (2σ) given the data from Tomkins *et al.* (orange dot and error bar in Fig. 3). The uncertainty is large because of the paucity of Archean micrometeorite data: Only two data points are available with metallic Fe fractional areas of 0.555 and 0.003, respectively (inferred from Tomkins *et al.*’s Fig. 1, E and F).

**Fig. 4:**
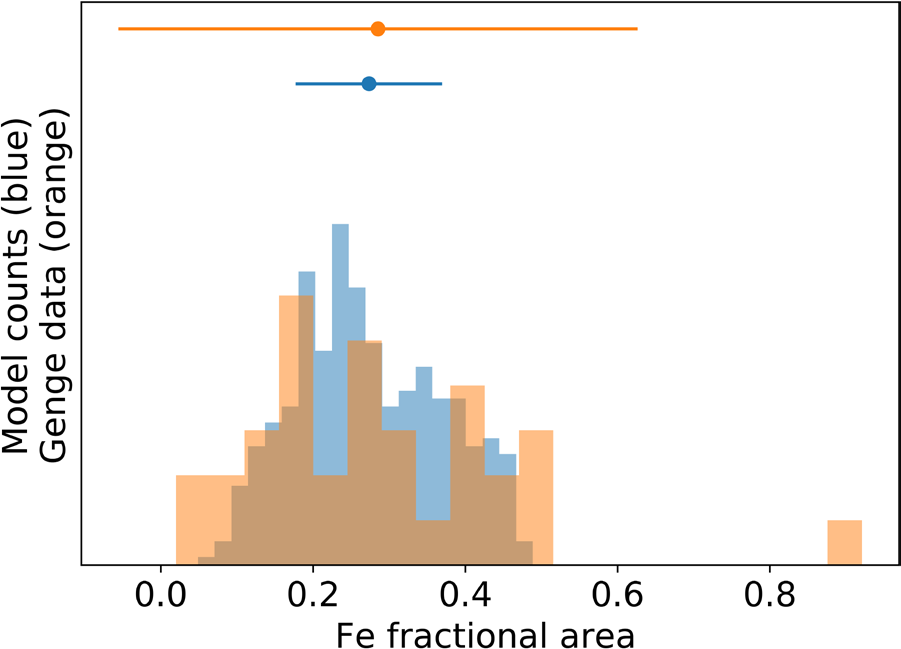
Fractional area of unoxidized Fe in simulated sectioned micrometeorites compared with observed modern micrometeorites. The horizontal axis in this plot shows the fractional area of unoxidized Fe in cross-sectioned micrometeorites. The blue histogram shows the simulated unoxidized Fe fractional area from 500 randomly generated micrometeorites entering the modern atmosphere, which were oxidized by O_2_, and the orange histogram shows the data inferred from figure 4 of Genge *et al.* (*15*). The model mean and 2σ confidence interval are shown by the blue dot and error bar, while the mean and 2σ of the data from Genge *et al.* are shown by the orange dot and error bar.

Some fully oxidized micrometeorites collected by Tomkins *et al.* indicate that high CO_2_ levels were likely present during entry. Our model predicts that fully oxidized micrometeorites will only form when atmospheric CO_2_ concentrations exceed ~70% by volume, as indicated by the dashed blue curve in Fig. 4. As such, while our CO_2_ estimate spans 6% to 100% (2σ), the CO_2_ concentration likely falls above ~70% given the presence of some fully oxidized micrometeorites in the Tomkins *et al.* data. This agrees with Archean paleosols and climate models of Archean Earth, which suggest that CO_2_ levels on the Archean were substantially higher than modern. If Archean Earth had a 1-bar atmosphere, then our estimate of >70% CO_2_ by volume indicates that the partial pressure of CO_2_ would be >0.7 bar in the Archean. However, surface pressure may have been much lower during the Archean, perhaps just 0.23 ± 0.23 bar (2σ) (*22*). With a mean surface pressure of 0.23 ± 0.23 bar, our model predicts a CO_2_ partial pressure of >0.16 ± 0.16 bar (2σ). Provided some methane was present, such as 0.5% (*30*), this thin, CO_2_-rich atmosphere could provide enough greenhouse warming to sustain liquid water under a faint young Sun [e.g., (*31*)]. Atmospheric methane would warm the surface during the Archean as a greenhouse gas but is not expected to interact with molten, Fe-rich micrometeorites (*10*) and thus should not alter their oxidation state.

In our model, the oxidation of micrometeorites in the upper atmosphere is not affected by the atmospheric surface pressure. Reducing the surface pressure would move the altitude at which micrometeorites oxidize closer to the surface (slightly altering the gravitational acceleration experienced by melting micrometeorites), but the overall pressures at which melting occurs do not appreciably change nor do our results. The difference in model results between a 1-bar atmosphere and a 0.23-bar atmosphere is negligible, which agrees with the findings of Tomkins *et al.*

## DISCUSSION

Our model shows that 2.7 billion–year–old micrometeorites (*10*) could have been oxidized by CO_2_ in a CO_2_-rich atmosphere. While our estimated CO_2_ abundance of $64_{-58}^{+36}$% (2σ) has large uncertainties, collection and analysis of additional Archean micrometeorites could greatly reduce the spread in this estimate. Using the parameterized climate model described in (*2*), ~70% CO_2_ for the Archean atmosphere, and an upper bound on Archean surface pressure of ~0.5 bar (*22*), the global mean surface temperature in the Archean would be ~30°C (or lower for lower total pressure), indicating a temperate Archean climate. An improved Archean CO_2_ measurement would allow more robust climate models to further refine the surface temperature of Archean Earth and the conditions, including pH of surface waters, in which early life evolved. Not only would this help address the hot versus temperate Archean debate, but it could also inform our understanding of Earth-like exoplanets and their potential habitability [e.g., (*32*)].

The uncertainty in our model output (gray-shaded region in Fig. 3) is more difficult to reduce. This uncertainty stems from the random sampling of our initial parameter distributions for mass, velocity, and entry angle, which have some inherent uncertainty on modern Earth (see Materials and Methods). Despite such uncertainties, our model can reasonably predict the Fe fractional area in modern micrometeorites entering an O_2_-rich atmosphere (e.g., Fig. 4).

The conclusion that a CO_2_-rich atmosphere can oxidize Fe micrometeorites is a reasonable alternative to oxidation via the O_2_-rich Archean atmospheres (*10*, *11*). As demonstrated by the metallurgy industry [e.g., (*27*)] and other laboratory measurements (*28*), CO_2_ can readily oxidize Fe, albeit less efficiently than O_2_. While we do not model oxidation past FeO, further oxidation is not precluded by our model as the oxide that forms during entry is a liquid Fe melt with dissolved oxygen [see (*14*) for a discussion]. If the melt acquires more O than Fe, then it could solidify as Fe_3_O_4_ rather than FeO. However, additional laboratory measurements of molten Fe beads quenching in simulated CO_2_-rich atmospheres are necessary to understand how CO_2_ oxidizes Fe liquids at high temperatures.

Our model results in Fig. 3 show that a >70% by volume CO_2_ atmosphere could fully oxidize micrometeorites and possibly produce magnetite. This is seen by the dashed blue line in Fig. 3, which shows the fraction of the total simulated micrometeorites that are fully oxidized after entry as a function of atmospheric CO_2_ abundance. Our simulation stops when a micrometeorite has no remaining unoxidized Fe, but such a micrometeorite encountering additional CO_2_ could continue to oxidize past FeO, possibly to Fe_3_O_4_ (see Materials and Methods).

In addition to showing that atmospheric CO_2_ could explain the observed, oxidized Archean micrometeorites, our model predicts that the average unoxidized Fe remaining in collected micrometeorites should decrease with increasing atmospheric CO_2_. From this relationship, it may be possible to constrain atmospheric CO_2_ concentrations not only in the Archean but also in the Proterozoic when atmospheric oxygen was scarce. Our CO_2_-N_2_-O_2_ atmosphere with 1% O_2_ in the upper atmosphere could be representative of the Proterozoic atmosphere (*29*) in which micrometeorite oxidation would still be dominated by CO_2_, as seen in Fig. 2 (orange curve). Numerous iron-rich micrometeorites have been found in Proterozoic rocks (*33*) and measuring the fractional area of unoxidized Fe in such samples could readily be done. Such measurements might provide a constraint on atmospheric CO_2_ at that time.

The CO_2_ concentration of the atmosphere is thought to have decreased over Earth’s history [e.g., (*2*)] from a major atmospheric constituent in the Archean to a few hundred parts per million today. If we compare that trend to the black curve in Fig. 3, we see that our model predicts the fractional area of unoxidized Fe in collected micrometeorites should increase with time—if the trend depends just on CO_2_ oxidation—from the Archean until the end of the Neoproterozoic, albeit with some uncertainty due to a possible O_2_ overshoot during the Great Oxidation Event. Oxidation by O_2_ might remain minor during the Proterozoic because the ground-level atmospheric O_2_ may have been ~0.2% absolute concentration or less (*29*) so the trend could hold (Fig. 2, orange curve). To verify this hypothesis, additional micrometeorites should be collected and analyzed.

## MATERIALS AND METHODS

### Experimental design

#### *Model description*

This work follows the models developed by Love and Brownlee (*34*) (hereafter LB) and Genge (*14*) (hereafter MG). The LB model describes the entry and evaporation of silicate micrometeorites on modern Earth. The MG model expands the model of LB to include Fe-rich micrometeorites and their oxidation during atmospheric entry. Below, we describe our model, which is an implementation of the MG model but for oxidation of Fe by CO_2_.

Following MG and LB, our model describes the motion, heating, evaporation, and oxidation of iron micrometeorites. We assume an initial velocity, *v* (m s^−1^), an initial mass, *m* (kg), and an initial entry angle from zenith θ with θ = 90^∘^ being tangential to Earth’s surface and θ = 0^∘^ indicating the particle is moving directly toward Earth’s surface. The motion of the particle in two dimensions, accounting for atmospheric drag, can be calculated via

$$\frac{\mathrm{dv}}{\mathrm{dt}}=g-\frac{3\rho_{a}v^{2}}{4\rho r}\hat{v}$$ (1)

where *g* is gravity (m s^−2^), *v* is velocity (m s^−1^), *t* is time (s), ρ_a_ is the atmospheric density (in kg m^−3^), ρ is the density of the micrometeorite (kg m^−3^), and *r* is the particle radius (m). The altitude, *a*, of the particle was assumed to start at 190 km above Earth’s surface, following LB, and tracked throughout the model. For a given altitude, *g* is easily calculated from $g={\mathrm{GM}}_{⊕}/r_{\text{alt}}^{2}$ for gravitational constant *G* = 6.67 × 10^−11^ N m^2^ kg^−2^ and Earth mass *M*_⊕_ = 5.97 × 10^24^ kg; here, *r*_alt_ is the radial distance from the center of Earth to altitude, *a*. The MSIS-E-90 Atmosphere Model (available at https://ccmc.gsfc.nasa.gov/modelweb/models/msis_vitmo.php) was used to generate an atmospheric density profile for modern Earth, which we used for both the modern and Archean atmospheres following (*35*). Even if the atmospheric pressure in the Archean were lower than modern (*22*), the density profile of the upper atmosphere generated by the MSIS-E-90 model would likely remain similar and produce similar model results, as noted by (*10*).

We used the MSIS-E-90 model to generate atmospheric densities, ρ_a_, and total atmospheric oxygen densities (both O and O_2_) at 1-km intervals from Earth’s surface to 190 km (see data file S1 atmosphere_data.txt for MSIS-E-90 input parameters and resulting data). We linearly interpolated between each data point to find the atmospheric density for a given altitude. When calculating atmospheric CO_2_ abundance in the model, we specify a CO_2_ wt %, and then multiply the wt % by ρ_a_. This was done to make conversions from the MSIS-E-90 density data to CO_2_ abundances a simple conversion. For example, if the model is run with 30 wt % CO_2_, then the atmospheric CO_2_ density will be found via 0.3ρ_a_, with ρ_a_ coming directly from the MSIS-E-90 data. When modeling CO_2_ atmospheres, we assume the remainder of the atmosphere is N_2_.

With the velocity of the micrometeorite known, the heat flux of the particle, *dq*/*dt*, in watts can be described by

$$\frac{\mathrm{dq}}{\mathrm{dt}}=\frac{\pi r^{2}\rho_{a}v^{3}}{2}-L_{v}\frac{{\mathrm{dm}}_{\text{evap}}}{\mathrm{dt}}-4\pi r^{2}\sigma T^{4}+\Delta H_{\text{ox}}\frac{{\mathrm{dm}}_{\text{ox}}}{\mathrm{dt}}$$ (2)

following equations 3 and 14 from MG. The first term on the right hand side of Eq. 2 describes the heat flux due to collisions with air, which incorporates the ram pressure, ρ_a_*v*^2^, experienced by the micrometeorite. The second term describes the heat flux due to evaporative mass loss with the latent heat of vaporization for both FeO and Fe given by *L_v_* = 6 × 10^6^ J kg^−1^ and the mass loss given by *dm*_evap_/*dt* (in kg s^−1^). The third term accounts for radiative heat loss with the Stefan-Boltzmann constant σ = 5.67 × 10^−8^ W m^−2^ K^−4^ and micrometeorite temperature *T* (in K). We assume a blackbody emissivity of unity for the radiative term, following MG. The final term describes the heat of oxidation of an Fe particle with *dm*_ox_/*dt* being the mass growth of the oxide layer (in kg s^−1^). From MG, Fe oxidation by oxygen is exothermic, with an oxidation enthalpy of Δ*H*_ox_ = 3.716 × 10^6^ J kg^−1^. Oxidation by CO_2_ is endothermic and has an oxidation enthalpy of Δ*H*_ox_ = − 4.65 × 10^5^ J kg^−1^.

Following MG, the Δ*H*_ox_ for CO_2_ is approximated from the standard enthalpies of formation at standard temperature and pressure. It is estimated for the reactants and products in

$$\text{Fe}+{\text{CO}}_{2}\to \text{FeO}+\text{CO}$$ (3)

where the energies for Fe, CO_2_, FeO, and CO are 0, −393.5 (*36*), −249.5 (*37*), and −110.5 kJ mol^−1^ (*37*), respectively. Putting these values into Eq. 3, we see that 33.5 kJ mol^−1^ is consumed in the reaction, or 4.65 × 10^5^ J kg^−1^ of FeO. The heat of oxidation has only minor impact on the model results, so we neglect the temperature dependence of Δ*H*_ox_ following MG. The Δ*H*_ox_ for CO_2_ is an order of magnitude smaller than for O_2_ in absolute value, so this assumption is especially reasonable for the CO_2_-rich atmosphere modeled here. It has been argued that the reaction described in Eq. 3 is the only plausible pathway of oxidation of Fe by CO_2_ under the conditions considered in this work, so we do not consider other Fe + CO_2_ products (*28*).

The heat flux can be related to the specific heat capacity, and to temperature via

$$\frac{\mathrm{dq}}{\mathrm{dt}}={\mathrm{mc}}_{\text{sp}}\;\text{and}\;\frac{\mathrm{dT}}{\mathrm{dt}}=\frac{\mathrm{dq}}{\mathrm{dt}}\cdot \frac{\mathrm{dT}}{\mathrm{dq}}$$ (4)

for mass *m* and specific heat of wüstite *c*_sp_ = 400 (J kg^−1^ K^−1^), which coats the molten Fe micrometeorite upon entry (*38*). As shown in MG, Eqs. 2 and 4 give an equation for rate of temperature change

$$\frac{\mathrm{dT}}{\mathrm{dt}}=\frac{1}{{\mathrm{rc}}_{\text{sp}}\rho }(\frac{3\rho_{a}v^{3}}{8}-\frac{3L_{v}}{4\pi r^{2}}\frac{{\mathrm{dm}}_{\text{evap}}}{\mathrm{dt}}-3\sigma T^{4}+\frac{3\Delta H_{\text{ox}}}{4\pi r^{2}}\frac{{\mathrm{dm}}_{\text{ox}}}{\mathrm{dt}})$$ (5)

Equation 5 is the same as equation 6 of MG, but with the heat of oxidation term included. We note that MG is missing a 3 in the 3σ*T*^4^ term, likely due to a typesetting error.

In our model, we assume that Fe is only oxidized to FeO and do not consider further oxidation, following MG, as the process of oxidation past FeO is uncertain. As such, we only consider the ratio of unoxidized Fe to oxidized Fe in micrometeorites that are not fully oxidized in our model results. Following MG, we assume that any liquid oxide that forms during melting is immiscible with the molten Fe core and coats the exterior of the micrometeorite. Thus, we can calculate the evaporative mass loss rate, *dm*_evap_/*dt*, by considering the rate of FeO (or Fe) evaporation using the Langmuir approximation, which is given by

$$\frac{{\mathrm{dm}}_{\text{evap}}}{\mathrm{dt}}=-4\pi r^{2}p_{v}\sqrt{\frac{M}{2\pi R_{\text{gas}}T}}$$ (6)

where *R*_gas_ = 8.314 J mol^−1^ K^−1^ is the ideal gas constant, *M* is the molar mass (0.0718 kg mol^−1^ for FeO or 0.0558 kg mol^−1^ for Fe), and *p*_v_ is the vapor pressure of the evaporating FeO or Fe (in Pa). The vapor pressure was determined experimentally by Wang *et al.* (*39*) and from MG is given by

$$\text{log}(p_{v})=10.3-20126/T$$ (7)

for FeO (note that Eq. 7 is the same as MG’s equation 13 but for units of Pa rather than dynes cm^−2^).

In addition to evaporation of the liquid oxide layer, our model allows the unoxidized Fe to evaporate as well. This is necessary because we consider low-CO_2_ atmospheres where the formation of a liquid oxide layer surrounding the micrometeorite can be slower than the rate of evaporation. To handle this in our model, at each time step we calculate the oxide mass loss rate via Eq. 6, and if it exceeds the total oxide mass remaining in the particle, we evaporate liquid Fe for the remainder of the time step. The liquid Fe evaporation is calculated from Eq. 6 as well, but *p*_v_ is defined by

$$\text{log}(p_{v})=11.51-1963/T$$ (8)

from the data in (*39*). Thus, the total evaporation can be given by

$$\frac{{\mathrm{dm}}_{\text{evap}}}{\mathrm{dt}}=\frac{{\mathrm{dm}}_{\text{evap}\_m}}{\mathrm{dt}}+\frac{{\mathrm{dm}}_{\text{evap}\_\text{ox}}}{\mathrm{dt}}$$ (9)

where *dm*_evap_m_/*dt* is the metallic Fe evaporated and *dm*_evap_ox_/*dt* is the Fe oxide evaporated via Eq. 6.

The final step is to track the mass of Fe metal and FeO oxide in the micrometeorite. In an oxygen-rich atmosphere, we assume the total oxygen accumulated by the micrometeorite is given by

$$\frac{\mathrm{dO}}{\mathrm{dt}}={\gamma \rho }_{O}\pi r^{2}v$$ (10)

where ρ_O_ is the total density of oxygen (both O and O_2_) encountered (in kg m^−3^), following MG. The γ term is a dimensionless factor between 0 and 1 that determines what fraction of the encountered oxidant is used to oxidize Fe (γ = 1 in this work, see MG for a discussion of γ and O_2_).

For oxidation by CO_2_, we calculate the reaction rate of Fe and CO_2_ from

$$r_{{\text{CO}}_{2}}=k[\text{Fe}][{\text{CO}}_{2}]$$ (11)

for rate constant *k* = 2.9 × 10^8^ exp(− 15155/*T*) m^3^ mol^−1^ s^−1^ from (*28*) with *r*_CO_2__ in mol m^−3^ s^−1^. The Fe concentration is given by

$$[\text{Fe}]=\frac{m_{\text{Fe}}}{V}$$ (12)

where *m*_Fe_ is the mass of Fe in the micrometeorite (in mol), and *V* is the volume of the micrometeorite (in m^3^). The CO_2_ concentration per unit volume is given by the total CO_2_ encountered per second multiplied by the time step, Δ*t*, i.e.

$$[{\text{CO}}_{2}]=\frac{{\gamma \rho }_{{\text{CO}}_{2}}\pi r^{2}v}{V}\Delta t$$ (13)

where ρ_CO_2__ is in mol m^−3^. We compare the rate in Eq. 11 to the total CO_2_ encountered per unit volume per time step and take the lesser of the two as the amount of oxygen accumulated by the Fe. We assume γ = 1 in Eq. 13, so the oxidation from CO_2_ calculated with this model should be considered an upper bound. From Eq. 3, each CO_2_ that reacts with the micrometeorite will add one O to the particle as FeO so

$$\frac{dO}{\mathrm{dt}}=V\cdot \text{min}(\frac{\left[{\text{CO}}_{2}\right]}{\Delta t},r_{{\text{CO}}_{2}})$$ (14)

When calculating *d*O/*dt* for CO_2_, we first calculate in mol s^−1^ and then convert to kg s^−1^ for ease of use in the model.

It is important to note that the reaction rate for Fe oxidation via CO_2_ used in this model was derived from laboratory measurements of gas-phase interactions of Fe and CO_2_ (*28*). This likely represents an upper bound on Fe oxidation via CO_2_ and may overestimate the oxidation of liquid Fe in a CO_2_-rich atmosphere, where diffusion of the oxidant through the liquid Fe oxide could be the rate-limiting step. However, the kinetics of the reaction for the pressures and temperatures considered in this model are uncertain (noting that temperatures often exceed ~2000 K). As such, the model presented here should be considered an upper bound on the oxidation rate by CO_2_. Future laboratory measurements are desirable to constrain the reaction rate described by Eq. 11.

Following MG, our model only allows oxidation, while unoxidized Fe remains in the micrometeorites. Thus, one Fe atom will be removed from the metallic Fe mass for each O atom accumulated. The total metallic Fe in the particle at each time step is then calculated by the amount Fe converted to Fe oxide, minus evaporated Fe, giving an equation for the mass of metallic Fe in the particle

$$\frac{{\mathrm{dm}}_{m}}{\mathrm{dt}}=-\frac{M_{\text{Fe}}}{M_{O}}\frac{dO}{\mathrm{dt}}-\frac{{\mathrm{dm}}_{\text{evap}\_m}}{\mathrm{dt}}$$ (15)

for Fe molar mass *M*_Fe_ = 0.0558 kg mol^−1^ and atomic O molar mass *M*_O_ = 0.0160 kg mol^−1^. Similarly, the mass of oxide will grow for each O atom encountered, minus the evaporated FeO, which can be described by

$$\frac{{\mathrm{dm}}_{\text{ox}}}{\mathrm{dt}}=\frac{M_{\text{FeO}}}{M_{O}}\frac{dO}{\mathrm{dt}}-\frac{{\mathrm{dm}}_{\text{evap}\_\text{ox}}}{\mathrm{dt}}$$ (16)

With initial altitude, velocity, and mass known, Eqs. 1, 5, 9, 15, and 16 can be solved numerically to simulate the entry of an Fe micrometeorite. We assume all particles start as pure Fe and use a simple Euler approximation to numerically integrate the equations. We set the maximum time step to 0.01 s for our integration but allow the time step to adjust dynamically such that the maximum change in temperature of the particle never exceeds 0.1%. Following MG, we assume that no oxidation occurs until the micrometeorite melts at 1809 K for Fe and oxidation shuts off when the micrometeorite solidifies at 1720 K (the FeO melting temperature). We assume the liquid FeO has a density of 4400 kg m^−3^ and Fe has a density of 7000 kg m^−3^, from MG. In addition, for Fe, we use a specific heat of 400 J K^−1^ kg^−1^. The Python script containing our model is available in the Supplementary Materials. Figure 1 shows an example model run for a single 50-μm particle entering a 50 wt% CO_2_, 50 wt% N_2_ atmosphere at 12 km s^−1^, and an entry angle of 45° from the Zenith.

Of interest in our model is the fractional area of unoxidized Fe compared with the total cross-sectional area of the micrometeorite (i.e., unoxidized Fe plus oxidized Fe) after they solidify. For a given micrometeorite, we assume that the unoxidized Fe forms a spherical bead at the center of the particle and is evenly surrounded by any produced oxide (FeO). We then “sectioned” the simulated micrometeorites at the midpoint and compared the total surface area of exposed metallic Fe to the total area of the sectioned micrometeorite. This quantity can be easily compared to measurements like those in figure 4 of (*15*), which reports Fe-phase abundance in sectioned micrometeorites. Our assumption that the metallic Fe is centered in the micrometeorite means we assume an upper bound on the sectioned area, as an uncentered bead may not measure Fe at the widest point. Despite this assumption, our model is able to accurately reproduce the data reported in figure 4 of (*15*), which shows the ratio of metallic Fe to oxidized Fe for 34 modern micrometeorites collected from Antarctica (we consider both FeO and Fe_3_O_4_ as oxides here and do not differentiate between them). Note that in (*15*), the captions of figures 4 and 5 are switched so the interested reader should look at the data presented in figure 4, but apply the caption of figure 5 to avoid confusion. Figure 4 in this paper shows our model prediction of Fe fractional area compared with the data inferred from figure 4 of (*15*). The agreement between our simulated data (blue) and the modern micrometeorite collected data (orange) is shown in the figure.

Following MG, we do not consider magnetite formation in our model because the process by which magnetite forms during entry is uncertain. The liquid Fe oxide that forms while the micrometeorite is molten could crystalize as Fe_3_O_4_ if enough oxygen is accumulated while molten. However, magnetite may also form after solidification due to decomposition of FeO to Fe_3_O_4_ or possibly from further oxidation at low temperatures. In addition, the central Fe bead could separate from the molten micrometeorite during entry, leading to a highly oxidized melt as a remainder, which could solidify as magnetite [see (*14*) for a discussion of Fe_3_O_4_ formation]. Thus, we only consider micrometeorites that still retain unoxidized Fe in both our model results and collected data and do not differentiate between the phases of oxidized Fe.

When calculating our simulated micrometeorite areas, we impose several conditions on the final particle. First, we do not consider simulated particles that are smaller than 2 μm in final radius. Despite the abundance of such small particles, they are not easily found when extracting micrometeorites from sedimentary rocks given their small size, so we do not consider them to better represent collected data. The smallest micrometeorite found by Tomkins *et al.* was ~4 μm in radius. Second, we do not consider fully oxidized or unoxidized micrometeorites (i.e., pure FeO or pure Fe). This is done as pure Fe micrometeorites only exist in our model because they enter the atmosphere slow enough, or with a shallow enough angle that they do not reach the Fe melting temperature. These unmelted micrometeorites represent a fixed feature in the model data that cannot inform of atmospheric composition. Pure FeO micrometeorites represent the limit of our model calculations, so aside from showing their production is expected from the model (dashed blue curve in Fig. 3), they are not considered when calculating the mean Fe fractional area (black/orange lines in Figs. 2 and 3). Neglecting these edge cases does not hinder our ability to reproduce modern micrometeorite data (Fig. 4) since we exclude fully oxidized micrometeorites from the collected data as well, ensuring our data sets are comparable.

To validate our model for modern Earth, we simulated 500 micrometeorites entering modern Earth’s O_2_-rich atmosphere. We compare the fractional unoxidized metallic Fe area of modeled micrometeorites to that of micrometeorites collected from Antarctica (*15*). The results of this comparison are shown in Fig. 4, with our simulated micrometeorites shown in blue and the modern micrometeorite data shown in orange. Our model is only defined while metallic Fe remains in the micrometeorites, so we compare only partially oxidized micrometeorites from both the data and our model. The agreement of the model mean and the data mean from (*15*) (Fig. 4) indicates that our model can predict the Fe fractional area in modern micrometeorites entering Earth’s O_2_-rich atmosphere.

### Initial micrometeorite distributions

Using the model described above, we randomly generated micrometeorites from initial mass, velocity, and impact angle distributions. These distributions are described in detail in Love and Brownlee (*34*) and summarized below. For the initial impact angle, values between 0° and 90° are valid. The fraction of particles with impact angle θ between θ_1_ and θ_2_ is given by *n*(θ_1_, θ_2_) = sin^2^θ_2_ − sin^2^θ_1_. The equivalent probability density function for θ is given by

$$P(\theta )=\text{sin}(2\theta ),0^{\circ }<\theta <90^{\circ }$$ (17)

which we use to randomly sample initial impact angles in this work. In our simulations, micrometeorites with initial impact angles greater than ~83° could “skip” off the top of the atmosphere. We stopped the simulation on such particles as soon as they began moving away from Earth to avoid long computation times. Only particles that continued into the atmosphere upon contact were calculated. Micrometeorites with initial angles greater than 83° represent less than 1.5% of the distribution, so the impact of neglecting such particles is likely small.

The initial velocity distribution is defined by λ(*v*, *v* + *dv*) = 1.791 × 10^5^*v*^−5.394^*dv* for velocity, *v* (in km s^−1^). The probability density function for *v* is then given by

$$P(v)=1.791\times 10^{5}v^{-5.394}$$ (18)

for *v* > 11.2 km s^−1^. In this work, we only consider initial velocities up to 20 km s^−1^. Initial velocities between 11.2 and 20 km s^−1^ represent over 92% of the distribution and is similar to the velocity limits used by both MG and LB. Micrometeorites entering the atmosphere faster than 20 km s^−1^ require an increasingly small time step to avoid numerical errors, which is detrimental to the overall runtime of the simulation. As such, we neglect unusual micrometeorites with velocities greater than 20 km s^−1^ and expect it should have little bearing on our final results.

The initial mass distribution of the cumulative number of particles of mass greater than *m* per square meter per second is defined by

$$F(m)={\left(2.2\times 10^{3}m^{0.306}+15\right)}^{-4.38}+1.3\times 10^{-9}{\left(m+10^{11}m^{2}+10^{27}m^{4}\right)}^{-0.36}$$ (19)

This function is defined at 1 AU for particles greater than ~10^−14^ g. We only consider masses between 2.346 × 10^−9^ and 2.932 × 10^−2^ g, which correspond to initial Fe particle radii of 2 and 1000 μm, respectively. The collected micrometeorites we compare to were all greater than 2 μm in radius, which is how we set the lower limit. Our model assumes that the micrometeorite radius is less than its mean free path in the upper atmosphere, which is only true for small particles. At 1000-μm radius, this assumption becomes less reasonable, and such large particles could develop a bow shock, which we do not consider. As such, our simulation of the largest particles (≥1000-μm radius) is uncertain. Fortunately, over 95% of the mass distribution results in particles with radii under 500 μm. Following Love and Brownlee (*34*), we normalize the above mass distribution between the mass range of interest to find the probability density function

$$P(m)=\frac{{\left(2.2\times 10^{3}m^{0.306}+15\right)}^{-4.38}+1.3\times 10^{-9}{\left(m+10^{11}m^{2}+10^{27}m^{4}\right)}^{-0.36}}{4.59811\times 10^{-13}}$$ (20)

for mass *m* in grams.

### Statistical analysis

For our analysis of model uncertainty, we assumed the model output was Gaussian. In addition, we assumed that the two data points available from Tomkins *et al.* formed a Gaussian and used them to calculate a mean and SD. The estimate from the Tomkins *et al.* data should be viewed with skepticism until additional data are collected. The code necessary to reproduce our analysis is included in the Supplementary Materials.

## Supplementary Material

http://advances.sciencemag.org/cgi/content/full/6/4/eaay4644/DC1

Download PDF

Data file S1

Movie S1

Atmospheric CO2 levels from 2.7 billion years ago inferred from micrometeorite oxidation

## SUPPLEMENTARY MATERIALS

Supplementary material for this article is available at http://advances.sciencemag.org/cgi/content/full/6/4/eaay4644/DC1 Data file S1. A zipped file containing our model as a Python script and the data files necessary to reproduce our results and figures. Movie S1. An animated version of Fig. 1. The movie also shows a simulated micrometeorite (gray sphere) and the corresponding micrometeorite cross section.
