# Supplementary material for: Atmospheric CO2 levels from 2.7 billion years ago inferred from micrometeorite oxidation
Source: Sci Adv. 2020 Jan 22;6(4):eaay4644. doi: 10.1126/sciadv.aay4644 (PMC6976288; doi:10.1126/sciadv.aay4644)
Supplement: http://advances.sciencemag.org/cgi/content/full/6/4/eaay4644/DC1 [file supp_6_4_eaay4644__index.html]

Science Advances | Science AdvancesAAASSearchScience AdvancesMenu

## Supplementary Materials

Download PDF

**Other Supplementary Material for this manuscript includes the following:**

- Data file S1 (.zip format). A zipped file containing our model as a Python script and the data files necessary to reproduce our results and figures.
- Movie S1 (.mp4 format). An animated version of Fig. 1. The movie also shows a simulated micrometeorite (gray sphere) and the corresponding micrometeorite cross section.

**Files in this Data Supplement:**

- Adobe PDF - aay4644\_SM.pdf
