# Supplementary material for: Atmospheric CO2 levels from 2.7 billion years ago inferred from micrometeorite oxidation
Source: Sci Adv. 2020 Jan 22;6(4):eaay4644. doi: 10.1126/sciadv.aay4644 (PMC6976288; doi:10.1126/sciadv.aay4644)
Supplement: Download PDF [file aay4644_SM.pdf]

Science Advances
AAAS

advances.sciencemag.org/cgi/content/full/6/4/eaay4644/DC1

Supplementary Materials for

Atmospheric CO_2 levels from 2.7 billion years ago inferred from micrometeorite oxidation

O. R. Lehmer*, D. C. Catling, R. Buick, D. E. Brownlee, S. Newport

*Corresponding author. Email: info@lehmer.us

Published 22 January 2020, *Sci. Adv.* **6**, eaay4644 (2020)
DOI: 10.1126/sciadv.aay4644

Other Supplementary Material for this manuscript includes the following:

(available at advances.sciencemag.org/cgi/content/full/6/4/eaay4644/DC1)

Data file S1 (.zip format). A zipped file containing our model as a Python script and the data files necessary to reproduce our results and figures.
Movie S1 (.mp4 format). An animated version of Fig. 1. The movie also shows a simulated micrometeorite (gray sphere) and the corresponding micrometeorite cross section.
